# Combination of Hypertension Along with a High Fat and Cholesterol Diet Induces Severe Hepatic Inflammation in Rats via a Signaling Network Comprising NF-κB, MAPK, and Nrf2 Pathways

**DOI:** 10.3390/nu9091018

**Published:** 2017-09-14

**Authors:** Yuan Yuan, Hisao Naito, Xiaofang Jia, Kazuya Kitamori, Tamie Nakajima

**Affiliations:** 1 College of Life and Health Sciences, Chubu University, 487-8501 Kasugai, Japan; yuankaede@yahoo.co.jp; 2 Department of Public Health, Fujita Health University School of Medicine, 470-1192 Toyoake, Japan; naitoh@med.nagoya-u.ac.jp; 3 National Institute for Nutrition and Health, Chinese Center for Disease Control and Prevention, 100050 Beijing, China; jiaxf2006@126.com; 4 College of Human Life and Environment, Kinjo Gakuin University, 463-8521 Nagoya, Japan; kitamori@kinjo-u.ac.jp

**Keywords:** hepatic inflammation, high-fat-cholesterol diet, hypertension, mitogen-activated protein kinase, nonalcoholic steatohepatitis, nuclear factor erythroid 2-related factor 2 pathway, nuclear factor-kappa B, spontaneously hypertensive rat, stroke-prone spontaneously hypertensive5/Dmcr, Wistar Kyoto

## Abstract

Populations with essential hypertension have a high risk of nonalcoholic steatohepatitis (NASH). In this study, we investigated the mechanism that underlies the progression of hypertension-associated NASH by comparing differences in the development of high fat and cholesterol (HFC) diet-induced NASH among three strains of rats, i.e., two hypertensive strains comprising spontaneously hypertensive rats and the stroke-prone spontaneously hypertensive 5/Dmcr, and the original Wistar Kyoto rats as the normotensive control. We investigated histopathological changes and molecular signals related to inflammation in the liver after feeding with the HFC diet for 8 weeks. The diet induced severe lobular inflammation and fibrosis in the livers of the hypertensive rats, whereas it only caused mild steatohepatitis in the normotensive rats. An increased activation of proinflammatory signaling (transforming growth factor-β1/mitogen-activated protein kinases pathway) was observed in the hypertensive strains fed with the HFC diet. In addition, the HFC diet suppressed the nuclear factor erythroid 2-related factor 2 pathway in the hypertensive rats and led to lower increases in the hepatic expression of heme oxygenase-1, which has anti-oxidative and anti-inflammatory activities. In conclusion, these signaling pathways might play crucial roles in the development of hypertension-associated NASH.

## 1. Introduction

Nonalcoholic steatohepatitis (NASH) is the advanced form of nonalcoholic fatty liver disease (NAFLD), and it is characterized by hepatocellular ballooning, inflammatory cell infiltration, and fibrosis, with a high probability of progression into hepatic cirrhosis and tumor, thereby leading to increased mortality compared with simple steatosis [1,2]. NAFLD/NASH is considered a hepatic manifestation of metabolic syndrome, and its development is frequently associated with hypertension, obesity, hyperlipidemia, and diabetes [1,3]. A high prevalence of NAFLD/NASH has been reported among patients with hypertension [4,5]. Hypertension has been shown to enhance the deterioration of NAFLD/NASH [6,7], but the mechanism that underlies the interaction between NASH and hypertension is still not understood completely.

Li et al. showed that a high fat (HF) diet induced modest hepatic steatosis in mice, while dietary cholesterol exacerbated liver damage and hepatic inflammation in mice fed with a HF diet [8]. Previously, we developed an animal model of NASH by feeding stroke-prone spontaneously hypertensive5/Dmcr (SHRSP5/Dmcr) rats with a high fat and high cholesterol (HFC) diet, where severe hepatic steatosis, ballooned hepatocytes, lymphocyte infiltrations, and fibrosis resembling human NASH were observed in the liver in this rat model after 8 weeks [9]. In this rat model, the HFC diet significantly elevated the serum level of the proinflammatory cytokine tumor necrosis factor-alpha (TNF-α) and activated its downstream signaling nuclear factor-kappa B (NF-κB) pathway, a central regulator of the inflammatory response, in the liver [10]. Moreover, in the absence of cholesterol, the HF diet only induced mild steatosis in our rat model, thereby suggesting the importance of cholesterol in NASH development (unpublished data).

SHRSP5/Dmcr rats are hypertensive, so the findings described above suggest that hypertension might influence the progression of HFC-induced fibrotic steatohepatitis by affecting inflammatory signaling pathways. SHRSP5/Dmcr, formerly known as arteriolipidosis-prone rats, are the fifth substrain of the stroke-prone spontaneously hypertensive rat (SHRSP) [9], which is derived from the spontaneously hypertensive rat (SHR) strain. The SHRSP5/Dmcr strain was produced by mating SHRSP rats, which have high serum cholesterol levels (about 600–900 mg/dL in females and 300–600 mg/dL in males) after feeding for one week with HFC diet. Repeated selective inbreeding led to gradual increases in the hypercholesterolemic responses of the offspring [9,11]. The new strain was developed as an animal model of arteriosclerosis, but marked enlargement and histopathological abnormities in the liver were found in the 47th generation [9]. The SHR strain was developed from normotensive Wistar strain rats [12]. Selected Wistar rats with spontaneously high systolic blood pressure (150 to 175 mmHg in males persisting for more than one month, and 130 to 140 mmHg in females) were mated, and the offspring with high blood pressure (over 150 mmHg persisting for more than one month) were selected for further inbreeding. The selection and inbreeding process was repeated, and all of the rats from the third generation to the sixth generation developed spontaneous hypertension within 15 weeks [12]. The SHR and Wistar Kyoto (WKY) rats were derived from the same parental outbred Wistar rats, so WKY rats have been used widely as the normotensive control for SHR rats, as well as SHRSP5/Dmcr. The blood pressure levels in the adult male WKY, SHR, and SHRSP5/Dmcr rats are 130 mmHg, 235 mmHg, and 180 mmHg, respectively [9]. Thus, both SHR and SHRSP5/Dmcr are hypertensive strains, whereas WKY is a normotensive strain.

In the pathogenesis of NAFLD/NASH, proinflammatory cytokines TNF-α and transforming growth factor-β (TGF-β) play essential roles in regulating the inflammatory response via mediating the activation of their downstream signaling pathways, such as NF-κB and mitogen-activated protein kinase (MAPK) [13,14,15,16]. On the other hand, oxidative stress has been implicated in the development of NAFLD/NASH [17]. The association between oxidative stress and blood pressure has been previously reported [18,19]. Therefore, nuclear factor erythroid 2-related factor 2 (Nrf2) signaling as well as its target gene heme oxygenase-1 (HO-1), which is important for antioxidant defense [20,21,22], may also be involved in the progression of hypertension-associated NASH.

In the present study, we investigated the roles of these pathways, NF-κB, MAPK, and Nrf2, as well as their interaction in the pathogenesis of hypertension-associated NASH using three strains, i.e., normotensive WKY, hypertensive SHR, and SHRSP5/Dmcr.

## 2. Materials and Methods

### 2.1. Animals and Experimental Protocol

**Figure 1:**
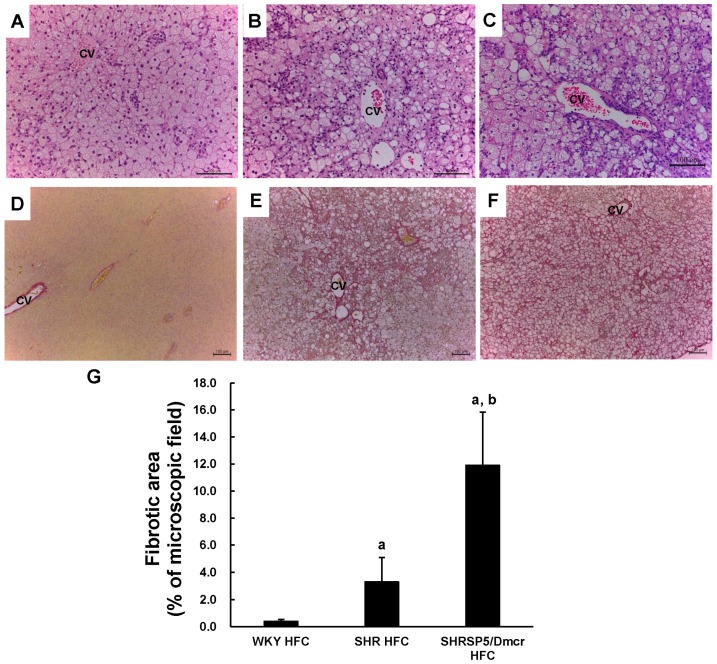
Representative images of liver sections subjected to staining with H&E (magnification: ×200 **(A**–**C**)) and EVG (magnification: ×100 (**D**–**F**)). Livers from Wistar Kyoto (WKY) rats fed the high fat and high cholesterol (HFC) diet for 8 weeks (**A**,**D**). Livers from spontaneously hypertensive rat (SHR) rats fed the HFC diet for 8 weeks (**B**,**E**). Livers from SHRSP5/Dmcr rats fed the HFC diet for 8 weeks (**C**,**F**); scale bar, 100 µm. CV, central vein. (**G**) fibrosis area (%), *n* = 6/group. ^a^ *p* < 0.05 vs. WKY HFC group; ^b^ *p* < 0.05 vs. SHR HFC group. H&E: hematoxylin and eosin; EVG: Elastic Van Gieson.

**Figure 2:**
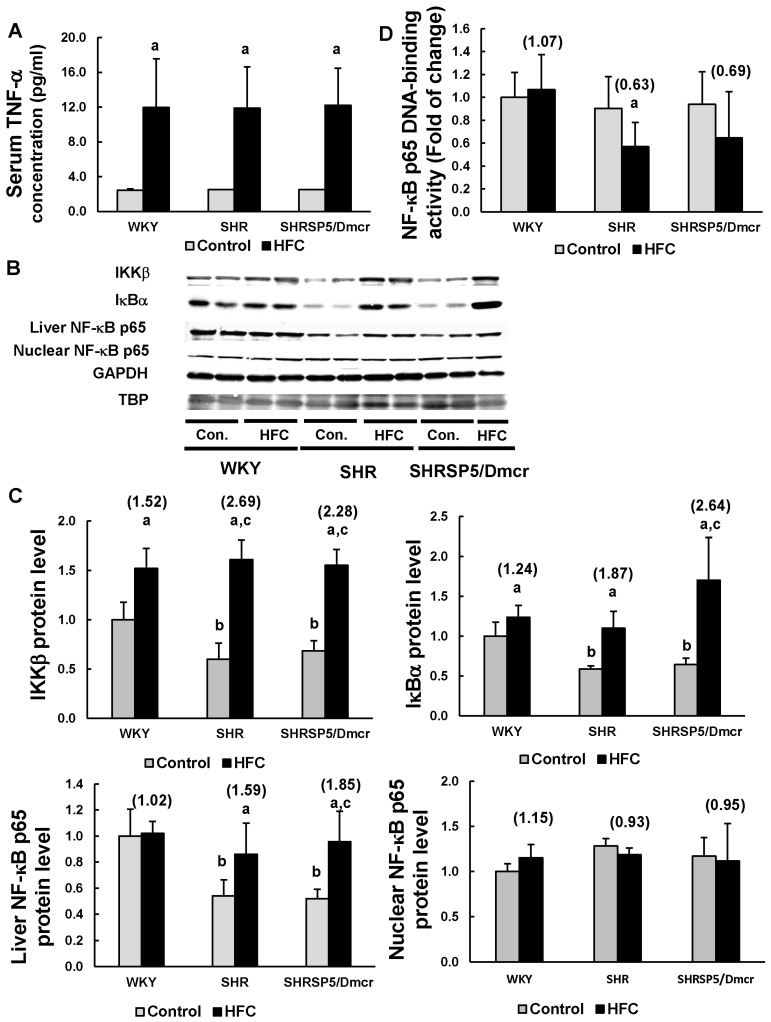
Effects of the HFC diet on the activation of the TNF-α/NF-κB pathway. ELISA analysis to measure the TNF-α serum levels in rats (**A**). Western blot analysis of the protein expression levels in liver homogenates or nuclear fragments (**B**,**C**). DNA-binding activity of NF-κB p65 (**D**). *n* = 6/group. The values in parentheses represent the fold changes compared with the respective control. ^a^ *p* < 0.05 vs. the respective control group; ^b^ *p* < 0.05 vs. WKY control group; ^c^ *p* < 0.05 vs. the fold change in WKY. TNF-α: tumor necrosis factor-α; IKKβ: inhibitor of κB kinase β; IκBα: inhibitor of κB-α; NF-κB: nuclear factor-κB; GAPDH: glyceraldehyde-3-phosphate dehydrogenase; TBP: TATA-binding protein; Con.: control.

**Figure 3:**
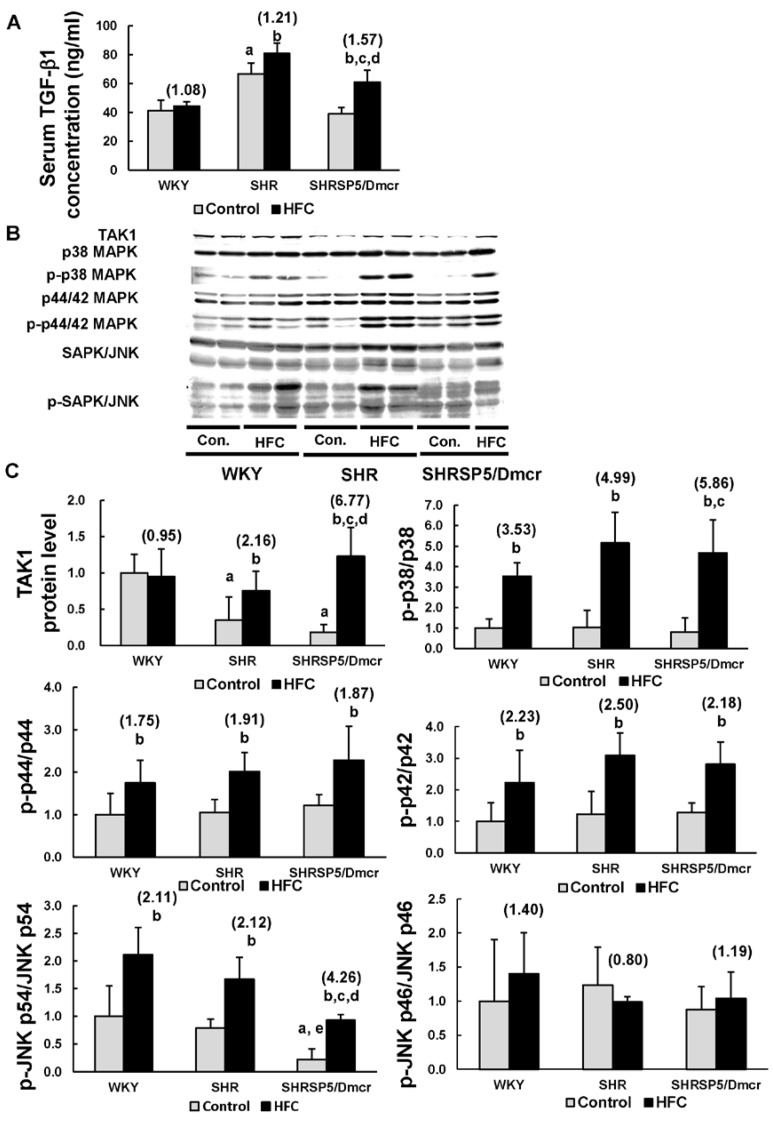
Effects of the HFC diet on the activation of the TGF-β1/MAPK pathway. ELISA analysis to measure the TGF-β1 serum levels (**A**). Western blot analysis of the protein expression levels in liver homogenates (**B**,**C**). *n* = 6/group. The values in parentheses represent the fold changes compared with the relative control. ^a^ *p* < 0.05 vs. WKY control group; ^b^ *p* < 0.05 vs. the respective control group; ^c^ *p* < 0.05 vs. the fold change in WKY; ^d^ *p* < 0.05 vs. the fold change in SHR; ^e^ *p* < 0.05 vs. SHR control group. TGF-β1: transforming growth factor-β1; TAK1: transforming growth factor beta-activated kinase 1; p38 MAPK: p38 mitogen-activated protein kinase; p-p38 MAPK: phosphorylated p38 MAPK; p44/42 MAPK: p44/42 mitogen-activated protein kinase; p-p44/42 MAPK: phosphorylated p44/42 mitogen-activated protein kinase; SAPK: stress-activated protein kinase; JNK: c-Jun N-terminal kinase; p-SAPK/JNK: phosphorylated SAPK/JNK; Con.: control.

**Figure 4:**
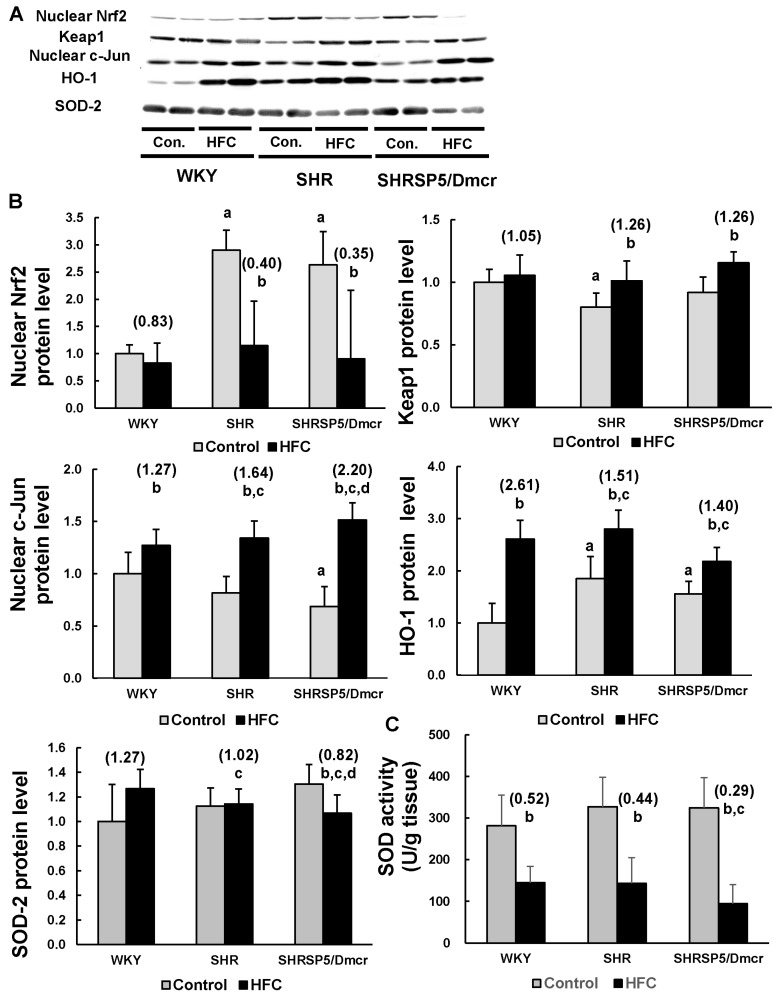
Effects of the HFC diet on the activation of the Nrf2 pathway and the SOD activity. Western blot analysis of the protein expression levels in liver homogenates or nuclear fragments (**A**,**B**). The total SOD activity (cytosolic and mitochondrial) was measured in the livers of the rats (**C**). *n* = 6/group. The values in parentheses represent the fold changes compared with the relative control. ^a^ *p* < 0.05 vs. WKY control group; ^b^ *p* < 0.05 vs. the respective control group; ^c^ *p* < 0.05 vs. the fold change in WKY; ^d^ *p* < 0.05 vs. the fold change in SHR. Nrf2: Nuclear factor erythroid 2-related factor 2; Keap1: Kelch-like ECH-associated protein 1; HO-1: heme oxygenase-1; SOD: superoxide dismutase; Con.: control.

All of the animal experiments were performed in accordance with the Guidelines for Animal Experiments at the Kinjo Gakuin University Animal Center (No. 65) and the Nagoya University Animal Center (No. 24247). WKY/Izm and SHR/Izm rats were obtained from Nippon SLC (Hamamatsu, Japan). Male and female SHRSP5/Dmcr rats with high serum cholesterol levels (100–300 mg/dL in males and 300–700 mg/dL in females) after one week of feeding with HFC diet were mated, and the male offspring were used in the present study. All of the rats used in the experiments were maintained under controlled conditions (23 ± 2 °C, 55 ± 5% humidity, 12/12 h light/dark cycle). Male rats aged 10 weeks from each strain were divided randomly into two groups (six rats in each group) and fed a HFC or control diet for 8 weeks before they were sacrificed. After fasting for 18 h, all of the rats were weighed and anesthetized using pentobarbital (70 mg/kg). Blood samples were collected and the serum was prepared by centrifugation at 3500× *g* for 10 min, before storing at −80 °C until use. The livers were harvested, weighed, and diced into small blocks. One small piece of each liver was fixed in 4% buffered paraformaldehyde, and the other parts were stored at −80 °C. The retroperitoneal side of the livers was used for most of the experiments (Figure 1, Figure 2, Figure 3 and Figure 4).

### 2.2. Biochemical Analyses

The serum levels of triglyceride (TG), total cholesterol (TC), aspartate aminotransferase (AST), alanine aminotransferase (ALT), and gamma-glutamyl transferase (GGT) were determined by SRL Inc. (Tokyo, Japan). Hepatic lipid was extracted according to the method described by Folch et al. [23]. Ten microliters (10 µL) of liver homogenate prepared by mixing 50 mg of liver tissue in 950 µL of 50 mM phosphate (K/K) buffer (pH 7.4) was added to 100 µL of chloroform–methanol (2:1) and centrifuged for 10 min at 10,000 g. The resultant chloroform phase (lower phase), which contained most of the tissue lipids, was used as a lipid source. The TG and TC levels in the liver samples were evaluated using TG-IE and T-Cho IE kits (Wako, Osaka, Japan), respectively.

### 2.3. Histopathology

The liver tissues fixed in 4% buffered paraformaldehyde were dehydrated using a graded ethanol series, treated with xylene and liquid wax, and finally embedded in paraffin. Tissue sections (4 µm) were prepared, which were stained with hematoxylin and eosin (H&E) or Elastic Van Gieson (EVG). Pathological conditions in the livers, such as microvesicular and macrovesicular steatosis, lobular inflammatory cell infiltration, hepatocyte ballooning, and fibrosis, were investigated under a DM750 microscope (Leica, Wetzlar, Germany) [9]. The fibrotic areas were quantified using NIS-Elements software (Nikon instruments, Tokyo, Japan) as described previously [24].

### 2.4. Western Blotting

Frozen liver tissues were minced finely and homogenized in 3 vol of ice-cold 0.25 M sucrose-10 mM phosphate buffer (pH 7.4), before centrifugation at 700× *g* for 10 min. The supernatant was collected as the liver tissue lysate. Nuclear proteins were extracted from liver tissues using a CelLytic^TM^ NuCLEAR^TM^ Extraction Kit (Sigma-Aldrich Japan, Tokyo, Japan). The samples were loaded on 10%-polyacrylamide-1% sodium dodecyl sulfate gels, electrophoresis was performed, and the samples were transferred onto polyvinylidene difluoride membranes (EMD Millipore, Billerica, MA, USA). The following antibodies were used to detect the corresponding proteins: inhibitor of κB-α (IκBα) and NF-κB p50 (Santa Cruz Biotechnology, Santa Cruz, CA, USA), inhibitor of κB kinase β (IKKβ), and NF-κB p65 (Cell Signaling Technology, Beverly, MA, USA); transforming growth factor beta-activated kinase 1 (TAK1), p38 mitogen-activated protein kinase (p38 MAPK), phosphorylated P38 MAPK (p-p38 MAPK), P44/42 MAPK (p44/42 MAPK), phosphorylated p44/42 MAPK (p-p44/42 MAPK), stress-activated protein kinase (SAPK)/c-Jun N-terminal kinase (JNK), and phosphorylated SAPK/JNK (P-SAPK/JNK) (Cell Signaling Technology, Beverly, MA, USA); Nrf2 (Santa Cruz Biotechnology, Santa Cruz, CA, USA), Kelch-like ECH-associated protein 1 (Keap1) and c-Jun (Cell Signaling Technology, Beverly, MA, USA), HO-1 (Abcam plc, Cambridge, UK), superoxide dismutase-2 (SOD-2) (Santa Cruz Biotechnology, Santa Cruz, CA, USA), and Cholesterol 7 alpha-hydroxylase (CYP7A1) (Abcam plc, Cambridge, UK). Western blotting was performed with antibodies against glyceraldehyde-3-phosphate dehydrogenase (GAPDH) (Santa Cruz Biotechnology, Santa Cruz, CA, USA) and TATA-binding protein (TBP) (Abcam plc, Cambridge, UK) as loading controls for liver homogenates and nuclear fractions, respectively. We used 1-Step^TM^ Ultra TMB-Blotting Solution (Pierce Biotechnology, Rockford, IL, USA) for signal development.

### 2.5. Enzyme-Linked Immunosorbent Assay (ELISA)

The serum levels of TNF-α and TGF-β1 were measured with Quantikine ELISA kits (R&D Systems, Minneapolis, MN, USA) according to the kit manufacturer’s recommended protocols. The NF-κB p65 DNA-binding activity was evaluated with a TransAM^TM^ NF-κB p65 transcription factor assay kit (Active Motif, Carlsbad, CA, USA) using the nuclear proteins extracted from rat livers.

### 2.6. SOD Activity Assay

After homogenizing the liver tissue, the total (cytosolic and mitochondrial) SOD activity was measured with a superoxide dismutase assay kit (Cayman Chemical, Ann Arbor, MI, USA) according to the kit manufacturer’s recommended protocols.

### 2.7. Statistical Analysis

Results were expressed as the mean ± standard deviation. The Student’s *t*-test was used to evaluate differences between the HFC-fed group and the corresponding control group. Fold changes were calculated as the ratio in the HFC group relative to the corresponding control group, and differences among the three strains of rats were determined using Tukey’s test. The protein level was expressed relative to the one of the WKY control group defined as 1.0. *p*-values < 0.05 were considered to indicate significant differences.

## 3. Results

### 3.1. Body and Liver Weights

The HFC diet led to lower body weights but higher liver weights compared with the control diet in all strains (Table 1). HFC feeding led to greater increases in the liver weight in the hypertensive strains, i.e., SHR (2.9-fold) and SHRSP5/Dmcr (3.4-fold) rats, compared with the normotensive strain, i.e., WKY rats (2.5-fold). A higher ratio of the liver weight relative to the body weight was observed in the hypertensive rats fed with the control diet, which suggests that abnormal enlargement of the liver occurred in the hypertensive strains before HFC administration. The HFC diet markedly elevated the ratio of the liver weight relative to the body weight in all three strains, but the increases in the hypertensive SHR (3.3-fold) and SHRSP5/Dmcr (3.7-fold) rats were greater than those in the normotensive WKY rats (2.8-fold). Interestingly, more significant changes in the liver weight and the ratio of the liver/body weight were observed in the SHRSP5/Dmcr rats fed with the HFC diet compared with the SHR rats, which suggests that the HFC diet may have caused more severe liver damage in the SHRSP5/Dmcr strain.

### 3.2. Serum and Hepatic Levels of Lipids and Liver Function Indices

In order to evaluate the lipid metabolism status following HFC feeding, we measured the serum and hepatic levels of TG and TC (Table 1). The serum level of TG was significantly higher in the hypertensive SHR and SHRSP5/Dmcr rats fed the control diet compared with the normotensive WKY rats, thereby suggesting that the accumulation of serum lipids occurred in the hypertensive rats before HFC feeding, which may have been attributable to the impaired metabolic function of the liver. The HFC diet did not affect the serum TG levels in the WKY and SHR rats, but they decreased slightly in the SHRSP5/Dmcr rats (0.7-fold). The hypertensive strains fed with the control diet had lower serum TC levels compared with the normotensive rats. The HFC diet markedly elevated the serum TC levels in all three strains, but a greater increase was observed in the SHRSP5/Dmcr rats (2.8-fold) compared with the WKY rats (1.4-fold). No significant differences in the hepatic levels of both TG and TC were observed among the three strains fed with the control diet. These levels were increased significantly by the HFC diet in all three strains. The increases in the hepatic TC levels induced by HFC in the SHR (42.8-fold) and SHRSP5/Dmcr (43.1-fold) rats were significantly lower than those in the WKY rats (51.8-fold), although HFC feeding led to similar increases in the hepatic TG levels in all three strains.

We also investigated the effects on the liver function of the HFC diet by determining the serum levels of AST, ALT, and GGT. The AST level was significantly higher in the SHR rats fed the control diet compared with those in the WKY and SHRSP5/Dmcr rats. The ALT levels were higher in both the SHR and SHRSP5/Dmcr strains fed the control diet, which indicated that some degree of liver injury occurred in the hypertensive rats in the absence of HFC feeding. The HFC diet increased the levels of AST and ALT in all three strains, but the increase in the AST level was greater in the SHRSP5/Dmcr rats (3.7-fold) than the WKY (2.5-fold) and SHR (2.0-fold) rats. HFC feeding led to similar increases in ALT levels in all three strains. There was no significant change in the GGT level in the WKY strain after feeding with the HFC diet, whereas it was markedly increased in the hypertensive rats. These results suggest that the HFC diet induced more severe liver damage in the hypertensive rats compared with the normotensive rats.

### 3.3. Histopathological Changes in the Liver

Liver sections stained with H&E were examined microscopically in order to investigate the histopathological changes induced by the HFC diet. Steatosis and hepatocyte degeneration were not observed in the three strains when fed the control diet. However, in the livers of WKY rats fed the HFC diet, microvesicular steatosis was clearly observed in zone 3 close to the central vein (CV) where oxygenation was poor compared with the portal triad region (Figure 1A). Mild lymphocyte infiltration and ballooned hepatocytes were observed, but there was no necrosis in the livers of this strain. However, macrovesicular steatosis, ballooned hepatocytes, and lymphocyte infiltration were much more evident in the livers of SHR rats fed the HFC diet (Figure 1B) compared with the WKY rats. Widespread necrosis was not observed in the livers in this strain. Similar to SHR rats, more severe steatosis and lymphocyte infiltration were observed in the livers of SHRSP5/Dmcr rats fed the HFC diet (Figure 1C), where macrovesicular steatosis was more predominant compared with microvesicular steatosis. In contrast to SHR rats, more ballooned hepatocytes with eosinophilic Mallory-Denk bodies were observed in SHRSP5/Dmcr rats, while hepatocyte necrosis was evident in wide-field views. Hepatic sinusoids around the CV were rarely observed in the livers of all three strains fed the HFC diet.

EVG staining was performed to assess liver fibrosis, and the fibrotic area was quantified. Fibrosis was not observed in the livers of all three strains fed the control diet. Fibrosis was also not observed in the HFC-fed WKY rats (Figure 1D), whereas moderate hepatic fibrosis occurred in the SHR rats following HFC feeding (Figure 1E). The severity of hepatic fibrosis in SHRSP5/Dmcr rats was obviously increased compared with that in SHR rats (Figure 1F). The results obtained by quantitative analysis were consistent (Figure 1G).

The HFC diet induced a more severe inflammatory response and fibrosis in the livers of the hypertensive rats (SHR and SHRSP5/Dmcr) compared with the normotensive rats (WKY), so we investigated the roles of inflammatory pathways in the development of HFC diet-induced liver damage. The mechanism that allows the HFC diet to induce hepatic fibrosis in hypertensive rats will be reported in the future.

### 3.4. TNF-α/ NF-κB Pathway

TNF-α is a critical pro-inflammatory cytokine that induces the activation of NF-κB pathway, which plays a crucial role in regulating inflammatory response [13]. Feeding with the HFC diet led to similar large increases in the serum TNF-α levels in all three strains (Figure 2A). The hepatic levels of IKKβ and IκBα, which are involved in the NF-κB pathway, were clearly lower in the hypertensive rats fed the control diet compared with those in the normotensive rats (Figure 2B,C). The HFC diet markedly increased the expression levels of both these proteins in the livers of all three strains. The increases in the IKKβ levels were greater in the SHR (2.69-fold) and SHRSP5/Dmcr rats (2.28-fold) than those in the WKY rats (1.52-fold), whereas the IκBα levels were greater in the SHRSP5/Dmcr rats (2.64-fold) compared with the WKY rats (1.24-fold). The NF-κB p65 levels in the livers of hypertensive rats fed with the control diet were markedly lower than those in the WKY rats. The HFC diet increased the NF-κB p65 levels in the hypertensive strains, but not in the normotensive rats, where the increased level was even greater in the SHRSP5/Dmcr rats (1.85-fold) compared with those in the SHR rats (1.59-fold). These findings suggest that NF-κB signaling was suppressed in the hypertensive strains before HFC feeding. Unexpectedly, there was no significant change in the nuclear level of NF-κB p65 in all three strains following HFC feeding. Furthermore, the HFC diet did not increase the DNA-binding activity of NF-κB p65 in all three strains (Figure 2D). However, the HFC diet did not affect the expression and nuclear accumulation of NF-κB p50 in the livers of all three strains (data not shown).

### 3.5. TGF-β1/MAPK Pathway

TGF-β1 has both pro-inflammatory and anti-inflammatory effects by activating different downstream signaling pathways, and it is also associated with liver fibrogenesis [14,25]. The hypertensive SHR rats fed the control diet exhibited higher TGF-β1 serum levels compared with the WKY rats (Figure 3A). The HFC diet markedly increased the serum level of TGF-β1 in the hypertensive rats, whereas no significant change was observed in the normotensive strain. The increases were greater in the SHRSP5/Dmcr rats (1.57-fold) compared with the SHR rats (1.21-fold). TGF-β1 activates TAK1, which is an essential mediator of inflammatory response, and it promotes the activation of downstream inflammatory signaling pathways, including the MAPK pathway [14]. Therefore, we determined the hepatic level of TAK1 and the proteins associated with MAPK pathways. The TAK1 level was markedly lower in the hypertensive rats in the control group than the normotensive rats (Figure 3B,C). The HFC diet induced the upregulation of TAK1 in the hypertensive rats, but it did not affect the expression levels in the normotensive rats. The increases in the TAK1 level were greater in the SHRSP5/Dmcr rats (6.77-fold) than the SHR rats (2.16-fold). The three classes of MAP kinases comprise p38 MAPK, p44/42 MAPK (ERK1/2, extracellular signal-regulated kinases), and JNKs [26]. The HFC diet significantly increased the phosphorylation of p38, p44, p42, and JNK p54 in all three strains. Furthermore, the increases in the phosphorylation of p38 and JNK p54 were more obvious in the SHRSP5/Dmcr rats than in the WKY rats. Compared with the SHR strain, the level of JNK p54 phosphorylation was lower in SHRSP5/Dmcr rats fed the control diet, whereas the HFC diet induced greater increases in the SHRSP5/Dmcr rats. The HFC diet did not change the level of JNK p46 phosphorylation in all three strains of rats. Therefore, the increased activation of TAK1-dependent p38 and JNK signaling might have partially contributed to the severe inflammation in the livers of hypertensive rats.

### 3.6. Nrf2/Keap1 Pathway

The Nrf2/Keap1 pathway plays important roles in protecting cells from various toxicants and stresses by regulating the expression of cytoprotective genes [20]. One of its target genes, HO-1, is the rate-limiting enzyme for heme degradation, and it exhibits cytoprotective effects against oxidative stress as well as an immunomodulatory capacity [21,22]. HO-1 expression is regulated by the Nrf2/ Keap1 pathway as well as c-Jun, which is a critical component of the AP-1 transcription factor, and it is activated by the JNK pathway [21,27]. The constitutive Nrf2 level in liver nuclei was much higher in the hypertensive rats than the normotensive rats (Figure 4A,B). The HFC diet did not affect the nuclear level of Nrf2 in the WKY rats, but the levels were markedly decreased in the SHR and SHRSP5/Dmcr rats. By contrast, the SHR rats in the control group had significantly lower levels of Keap1, which is the inhibitor of Nrf2, compared with the WKY rats. Keap1 was upregulated after feeding with HFC in the SHR and SHRSP5/Dmcr rats, but not in the WKY rats. Furthermore, the nuclear level of c-Jun was slightly lower in SHRSP5/Dmcr rats fed the control diet than in the WKY strain. The HFC diet increased the c-Jun levels in all three strains, but the increases were greater in the hypertensive rats. The increases in the c-Jun levels were significantly higher in the SHRSP5/Dmcr rats (2.20-fold) than the SHR rats (1.64-fold). Interestingly, higher hepatic levels of HO-1 were observed in the hypertensive rats fed the control diet compared with the normotensive strain. HO-1 was upregulated following HFC feeding in all three strains, where the fold increase was lower in the hypertensive strains (1.51-fold in SHR rats and 1.40-fold in SHRSP5/Dmcr rats) compared with the normotensive rats (2.61-fold).

SOD is also located downstream of the Nrf2 pathway, and it plays essential role in antioxidant defense by eliminating superoxide radicals [28]. The HFC diet decreased the SOD-2 level in the livers of the SHRSP5/Dmcr strain, but it tended to increase its expression in the WKY rats (Figure 4A,B). The total SOD activity was reduced in all three strains following HFC feeding, but a more significant decrease was observed in the hypertensive SHRSP5/Dmcr rats compared with the normotensive rats (Figure 4C).

### 3.7. CYP7A1

**Figure 5:**
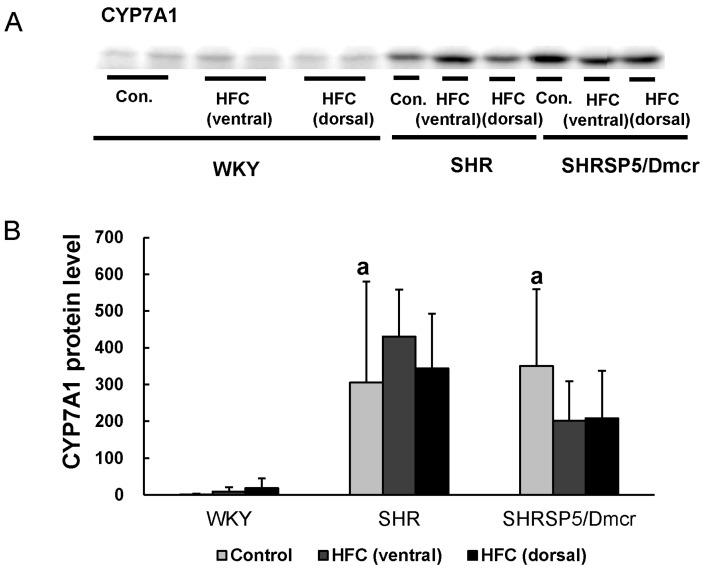
Differences in the constitutive level of hepatic CYP7A1, an important enzyme for bile acid synthesis, among the three strains. Western blot analysis in liver homogenates (**A**,**B**). *n* = 6/group. ^a^ *p* < 0.05 vs. WKY control group. CYP7A1: cholesterol 7 alpha-hydroxylase; Con.: control.

In a previous study, we showed that dysregulated bile acid synthesis may be implicated in HFC-induced fibrotic steatohepatitis progression in SHRSP5/Dmcr rats [29]. Therefore, we determined the protein expression level of CYP7A1, which is the important enzyme involved in bile acid synthesis, in the ventral and retroperitoneal (dorsal) sides of the livers of the three strains [30] (Figure 5A,B). The constitutive CYP7A1 level was much higher in the two hypertensive stains than in the normotensive one. The HFC diet did not affect the protein expression of CYP7A1 in WKY and SHR rats. Meanwhile, it tended to decrease the CYP7A1 level in the livers (ventral and dorsal) of SHRSP5/Dmcr rats, although no statistical significance was observed. These results suggested that the upregulation of CYP7A1 in the hypertensive strains may also contribute to the progression of hypertension-associated NASH, by affecting bile acid metabolism.

## 4. Discussion

In this study, our biochemical and histopathological analyses showed that the HFC diet induced a more severe inflammatory response and fibrosis in the livers of the hypertensive rats (SHR and SHRSP5/Dmcr) compared with the normotensive rats (WKY). The results obtained for the SHRSP5/Dmcr rats were mostly the same as those reported previously [9,10], but HFC-induced hepatitis and fibrosis were even more severe in the SHRSP5/Dmcr rats compared with the SHR strain. The molecular mechanisms responsible for differences in the HFC-induced inflammatory response in the hypertensive and normotensive strains might be related to the following pathways: (1) stronger activation of proinflammatory TGF-β1/MAPK signaling and the NF-κB pathway (IKKβ and IκBα) in the hypertensive strains compared with the normotensive strain; and (2) suppression of the Nrf2/Keap1 pathway in hypertensive rats increasing the hepatic expression of HO-1 compared with normotensive rats. These findings suggest that hypertension is one of the crucial factors related to HFC-induced NASH development, and the signaling pathways mentioned above may be important for the pathogenesis of hypertension-associated NASH. We found that the two hypertensive strains exhibited abnormal enlargement and mild injury in their livers before HFC feeding, as shown by the lower serum TC levels and higher ALT levels in both the hypertensive strains as well as by the higher serum AST levels only in the SHR rats, which suggests that some intrinsic factors associated with hypertension may have affected the development of hepatic inflammation.

Inflammation is one of the most important features of NASH [31], and it is associated with subsequent pathological progress (hepatic fibrosis, cirrhosis, and hepatocellular carcinoma) [32,33,34]. The release of proinflammatory cytokines, including TNF-α, from activated Kupffer cells is critical for triggering the inflammatory response during the development of NASH [35]. TNF-α expression is upregulated in the livers of NASH patients, and its level is associated with pathological severity [36,37]. TGF-β is a pleiotropic cytokine, which is implicated in both inflammation and fibrosis in chronic liver diseases [25]. The present study showed that the serum TNF-α and TGF-β1 levels were both elevated in the hypertensive strains following HFC feeding, whereas there was no increase in TGF-β1 in the normotensive strain. Therefore, the combination of these cytokines may have initiated more severe inflammatory responses in the hypertensive rats by activating the downstream signaling of NF-κB and MAPK. In addition, TAK1 is important for signaling transduction following TGF-β activation [38,39]. The HFC diet increased the hepatic TAK1 levels only in the hypertensive strains, where the increase was higher in the SHRSP5/Dmcr rats than the SHR rats. Furthermore, the HFC diet induced greater increases in the phosphorylation of p38 and JNK p54, which are involved with MAPK signaling, in the hypertensive SHRSP5/Dmcr strain compared with the normotensive WKY, thereby suggesting that the increased activation of TAK1-dependent MAPK signaling (p38 and JNK) may have contributed to severe hepatic inflammation in the hypertensive rats.

In addition, the activation of the NF-κB pathway mediated by the cytokines TNF-α and TGF-β1 is observed commonly in human and animal NASH models [13,15,16]. In the present study, the HFC diet induced higher increases in the protein expression levels of IKKβ, IκBα, and NF-κB p65 in the livers of hypertensive rats compared with the normotensive strain, though it did not increase the nuclear accumulation of NF-κB p65 or the NF-κB p65 DNA-binding activity in the three strains. Our previous study showed that the nuclear level of NF-κB p65 increased markedly in the livers of hypertensive SHRSP5/Dmcr rats after the administration of HFC for 2 weeks, but no significant increases were observed at 8 and 14 weeks [10]. Therefore, we consider that the NF-κB pathway might be activated by the HFC diet during the initial stage of inflammation (2 weeks), but the activation appeared to be discontinued in the following stage (8 and 14 weeks). Interestingly, the hepatic levels of IKKβ, IκBα, and NF-κB p65 were significantly lower in the hypertensive strains fed the control diet compared with the normotensive rats, thereby suggesting that this signaling was repressed before HFC feeding. Therefore, hypertension itself might facilitate the production of a genetic/epigenetic context that is more susceptible to proinflammatory stimuli by changing the expression levels of proteins involved in the NF-κB pathway.

The role of oxidative stress in NASH pathogenesis is well-established, where it is increased in patients with NASH [40,41]. HO-1 is a stress-inducible protein, and it plays a protective role in oxidant-induced injury [21,22], where it catalyzes the degradation of pro-oxidant and pro-inflammatory heme, and induces the production of antioxidant and anti-inflammatory bilirubin [21]. Increased HO-1 expression levels are observed in NASH patients [42]. In the present study, we showed that the HFC diet increased the hepatic level of HO-1 in all three strains, but the fold increase was lower in the hypertensive rats than the normotensive rats. The expression of HO-1 is regulated by several pathways, including the Nrf2/Keap1 and JNK/c-Jun pathways [21]. The HFC diet suppressed the nuclear accumulation of the transcriptional factor Nrf2 in the hypertensive strains, but the expression of its inhibitor Keap1 was increased. The HFC diet did not affect this pathway in the normotensive rats. Feeding with the HFC diet increased the nuclear level of c-Jun in all three strains, but the increases were greater in the hypertensive rats. Thus, the Nrf2/Keap1 and MAPK/c-Jun pathways coregulated the expression of HO-1 and reduced the levels of HO-1 in the hypertensive strains following HFC feeding compared with the normotensive strain. Interestingly, the levels of Nrf2 and HO-1 were markedly higher in the hypertensive rats fed the control diet than in the normotensive rats, thereby indicating the abnormal redox status of the livers in the hypertensive rats. In addition, the Nrf2 and NF-κB pathways are considered to inhibit each other [43,44]. Nrf2 signaling inhibits the NF-κB pathway by increasing the expression levels of proteins involved with antioxidant defense, thereby decreasing the activation of NF-κB mediated by reactive oxygen species. Therefore, the high level of Nrf2 in the hypertensive strains fed with the control diet may have been responsible for the low levels of proteins involved in the NF-κB p65 pathway.

SOD also plays an important role in antioxidant defense by catalyzing the dismutation of superoxide anions [28]. In our previous study, we demonstrated that the HFC diet decreased the hepatic level of SOD1 in the hypertensive SHRSP5/Dmcr strain [45]. In the present study, we also found that SOD2 expression was suppressed by the HFC diet in the livers of SHRSP5/Dmcr rats but not in those of the WKY and SHR rats. Furthermore, HFC feeding reduced the SOD activity in all three strains, but a greater decrease was found in the hypertensive SHRSP5/Dmcr strain. Thus, the great decrease in the SOD activity and the suppressed expression of HO-1 meant that the antioxidant capacity was highly attenuated in the SHRSP5/Dmcr strain, whereas the reduced expression of HO-1 only occurred following HFC feeding in the SHR rats.

Furthermore, in contrast to the SHR rats, more severe hepatic inflammation was observed in SHRSP5/Dmcr rats, as shown by the AST level and fibrosis, which may have been attributable to the greater activation of proinflammatory signaling (TGF-β1, TAK1, and JNK p54). There were also differences in HFC-induced NASH between the two hypertensive strains, i.e., SHR and SHRSP5/Dmcr, which might have been associated with different breeding selection methods, where the former was produced by mating Wistar strain rats with spontaneously high systolic blood pressure in normal condition, and the latter strain was developed from selected SHPSP rats with increased hypercholesterolemic responses following one week of feeding on the HFC diet [11,12]. Further studies should try to explain these differences in the future.

Hypertension is considered to be associated with oxidative stress [18,19]. Several antihypertensive agents have been reported to reduce oxidative stress [18,46,47], which suggests that oxidative stress may be a consequence of hypertension. Nrf2 has been implicated in the regulation of blood pressure [48], and one of its targets, HO-1, has a hypotensive effect when it is upregulated in animal models of hypertension [49,50]. HO-1 induces the production of CO by heme degradation, which has a vasodilatory effect and it inhibits the production of endothelin, a vasoconstrictor [48]. Therefore, compared with the normotensive rats, the higher levels of Nrf2 in the hypertensive strains fed the control diet, as well as the suppression of its activation following HFC feeding, may contribute to the pathogenesis of hypertension-associated NASH in our animal models.

**Figure 6:**
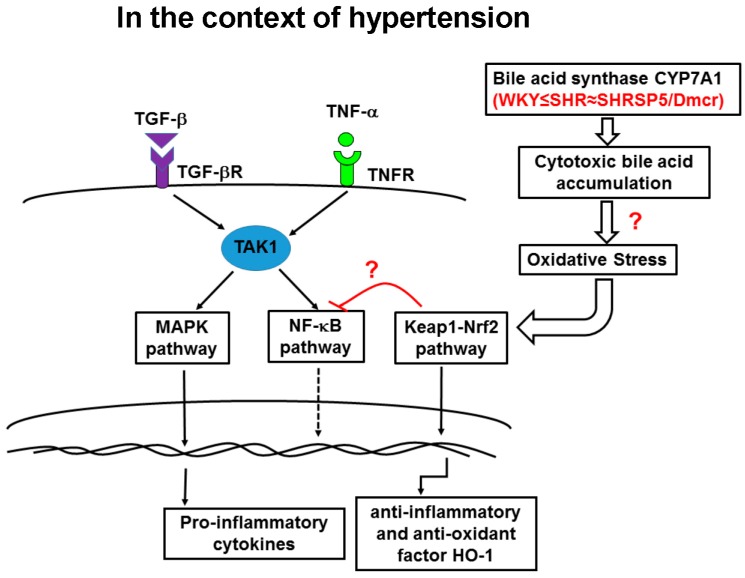
Interactions among the MAPK, NF-κB, and Nrf2 signaling pathways contribute to the progression of HFC diet-induced NASH in the context of hypertension. The inflammatory cytokines, TGF-β1 and TNF-α, bind to their receptors and activate downstream signaling in the MAPK pathway, as well as NF-κB via TAK1, following HFC feeding, which may be responsible for the severe inflammatory response in the livers of the hypertensive strains. Compared with the normotensive rats, the HFC diet induced increased activation of the MAPK pathway in the hypertensive strains. The NF-κB pathway was activated in the initial stage of inflammation (after 2 weeks feeding with the HFC diet), but the activation appeared to be discontinued for unknown reasons in the following stage (8 and 14 weeks). The Nrf2 pathway was repressed following HFC feeding, which suppressed the upregulation of the anti-inflammatory and antioxidant factor HO-1, thereby suggesting that the antioxidant capacity was attenuated in the hypertensive strains. In addition, an overexpression of the bile acid synthase CYP7A1 in the hypertensive strains may lead to the accumulation of cytotoxic bile acids in the livers and cause oxidative stress, thereby inducing mild inflammatory response and activating the Nrf2/Keap1 pathway before feeding the HFC diet. Therefore, the combination of increased activation of the inflammatory signaling pathways and suppression of the antioxidant pathway after HFC feeding may have contributed to the exacerbation of hepatitis.

In this study, we proposed a network of signaling pathways that contribute to the progression of HFC diet-induced NASH in hypertensive strains by considering previous research [13,14,16,21,25,26] (Figure 6). In the context of hypertension, the higher level of nuclear Nrf2 or the lower level of TAK1 may have been associated with the lower expression of proteins involved in the NF-κB pathway in the absence of HFC diet. Therefore, the hypertensive rats may have exhibited higher susceptibility to inflammatory stimuli. Following HFC administration, the increased activation of the MAPK (JNK and p38) signaling pathway was responsible for more severe inflammation in the hypertensive strains compared with the normotensive strain. The NF-κB pathway was implicated in regulating the inflammatory response in the initial stage, but its activation was soon discontinued. The HFC diet also induced the suppression of the Nrf2 pathway, and there was a greater decrease in the SOD activity in the hypertensive rats. Therefore, the hypertensive strains appeared to have a lower antioxidant capacity, and they were more susceptible to oxidative damage compared with the normotensive strain.

Our previous study showed that the accumulation of bile acid might occur in the livers of SHRSP5/Dmcr rats when fed the HFC diet, which may lead to oxidative stress [29]. Thus, it would be useful to identify the hepatic levels of bile acids after feeding with a HFC diet in the three strains in future research. Importantly, we found that the hepatic expression of CYP7A1 was over 300-fold higher in the hypertensive strains when fed the control diet compared with the normotensive rats. CYP7A1 is a rate-limiting enzyme that participates in the initial stage of bile acid synthesis, and it catalyzes the production of primary bile acids [30]. Kamisako et al. showed that Nrf2 may regulate the expression of genes implicated in fatty acid metabolism and bile acid synthesis, including CYP7A1 [51]. Moreover, CYP7A1 overexpression may lead to the excessive accumulation of toxic bile acids, such as hydrophobic bile acids [52,53]. Therefore, the increase in the Nrf2 activity may have led to the upregulation of CYP7A1 and a greater accumulation of toxic bile acids, thereby exacerbating the inflammatory responses in the hypertensive strains. The primary bile acids are synthesized in the liver via the oxidation of cholesterol [52]. In the present study, we showed that the serum level of TC was significantly lower in the hypertensive strains fed the control diet compared with the normotensive strain. A lower increase in hepatic TC was also found in the hypertensive strains following feeding with the HFC diet. Consistent with these results, Sandra et al. showed that the serum TC level was higher in CYP7A1 knockout mice compared with the wild-type strain. Therefore, increased bile acid synthesis might have occurred due to the increased expression of CYP7A1 in the hypertensive strains [54].

## 5. Conclusions

In the context of hypertension, a signaling network comprising the NF-κB, MAPK, and Nrf2 pathways plays critical roles in the pathogenesis of HFC diet induced-NASH by mediating increases in the inflammatory response and attenuating the antioxidant capacity.

## Figures and Tables

**Table 1:** Body and liver weights, and serum and hepatic levels of various biochemical indices in the rats.

Rat strains	WKY		SHR		SHRSP5/Dmcr	
	Control	HFC	Control	HFC	Control	HFC
Body weight (g)	365 ± 22.6	331 ± 7.8 ^a^	339 ± 14.5	301 ± 10.8 ^a^	283 ± 16.4	260 ± 9.1 ^a^
Liver weight (g)	9.1 ± 0.6	23.2 ± 1.4 ^a^ (2.5)	9.8 ± 0.4	28.5 ± 2.5 ^a^ (2.9) ^b^	8.0 ± 0.4	27.4 ± 1.8 ^a^ (3.4) ^b,d^
Liver/body weight(%)	2.5 ± 0.1	7.0 ± 0.3 ^a^ (2.8)	2.9 ± 0.0 ^c^	9.5 ± 0.8 ^a^ (3.3) ^b^	2.8 ± 0.1 ^c^	10.6 ± 0.8 ^a^ (3.7) ^b,d^
Serum						
TG (mg/dL)	12.3 ± 1.9	12.2 ± 2.6(1.0)	17.7 ± 3.3 ^c^	17.0 ± 2.8(1.0)	25.5 ± 4.1 ^c^	17.7 ± 4.6 ^a^ (0.7) ^b,d^
TC (mg/dL)	103 ± 7.8	140.3 ± 17.4 ^a^ (1.4)	61.3 ± 5.4 ^c^	134 ± 19.6 ^a^ (2.2)	53.5 ± 2.6 ^c^	149.2 ± 50.1 ^a^ (2.8) ^b^
AST (IU/L)	168.7 ± 47.6	424 ± 94.5 ^a^ (2.5)	245.7 ± 49.1 ^c^	479.2 ± 93.9 ^a^ (2.0)	177.8 ± 30.6 ^e^	633.8 ± 81.8 ^a^ (3.7) ^b,d^
ALT (IU/L)	33.8 ± 1.7	134.2 ± 50.0 ^a^ (4.0)	51.7 ± 6.3 ^c^	205.7 ± 47.5 ^a^ (4.0)	61 ± 11.0 ^c^	318.5 ± 55.0 ^a^ (5.2)
GGT (IU/L)	1.5	1.8 ± 0.6(1.2)	1.5	3.6 ± 1.6 ^a^ (2.4)	1.5	4.8 ± 1.0 ^a^ (3.2)
Liver						
TG (mg/dL)	23.9 ± 3.1	64.6 ± 9.4 ^a^ (2.7)	29.6 ± 7.1	63.6 ± 8.0 ^a^ (2.1)	26.8 ± 7.3	68.6 ± 15.8 ^a^ (2.6)
TC (mg/dL)	3.4 ± 0.4	176.2 ± 9.1 ^a^ (51.8)	3.9 ± 0.6	167 ± 10.7 ^a^ (42.8) ^b^	3.5 ± 1.0	150.9 ± 15.4 ^a^ (43.1) ^b^

Data represent the mean ± SD (*n* = 6); *p* < 0.05 indicates a significant difference; fold changes were calculated as the ratio in the HFC diet-fed group relative to that in the control group (numbers in parentheses); ^a^ *p* < 0.05 vs. the corresponding control group; ^b^ *p* < 0.05 fold changes in SHR or SHRSP5/Dmcr compared with that in WKY; ^c^ *p* < 0.05 vs. WKY control group; ^d^ *p* < 0.05 fold changes in SHRSP5/Dmcr compared with that in SHR; ^e^ *p* < 0.05 vs. SHR control group; TG: triglyceride; TC: total cholesterol; AST: aspartate aminotransferase; ALT: alanine aminotransferase; GGT: gamma-glutamyl transferase.
